# Metastatic retinoblastoma of the parotid and submandibular glands: a rare case report

**DOI:** 10.1186/s12886-017-0627-8

**Published:** 2017-12-02

**Authors:** Ping Wang, Yang-Jun Li, Shao-Bo Zhang, Qi-Lin Cheng, Qiong Zhang, Li-Sha He

**Affiliations:** Department of Ophthalmology, Tangdu Hospital of Fourth Military Medical University, Xi’an, Shaanxi China

**Keywords:** Parotid, Submandibular glands, Retinoblastoma, Metastasis, Treatment

## Abstract

**Background:**

Retinoblastoma is the most common intraocular malignancy occurring in children. It can metastasize to the regional lymph nodes, central nervous system and distant organs usually the bones and bone marrow and very rarely to the soft tissue. Here, we report a case of unilateral retinoblastoma in a 4-year-old girl accompanied by a large metastasis of the parotid and submandibular glands that developed about 6 months previously and gradually increased in size 5 months after enucleation of the left eye.

**Case presentation:**

A 4-year-old girl with a history of unilateral retinoblastoma presented with a large, painful and worsening mass (about 20 × 23 cm) of the left side of the neck. Following surgery, the orbital tumour was completely resected, and the large tumour invasion range in the left side of the neck was not resected completely. Histopathological examination revealed retinoblastoma of the orbit and the parotid and submandibular glands. After chemotherapy and additional local radiotherapy on the parotid and submandibular glands, the tumour was inactive and stable.

**Conclusions:**

Delayed detection and inappropriate management contribute to poor outcomes. Fundus examinations, education regarding the early signs of RB, and optimization of the therapeutic strategy for RB may play important roles in ocular health.

## Background

Retinoblastoma is the most common intraocular malignancy in children, with a reported incidence ranging from 1 in 15,000 to 1 in 18,000 live births [1]. Orbital retinoblastoma is one of the major contributors to mortality and carries a poor prognosis for life [2–5]. The frequency of metastatic retinoblastoma ranges from 4.8% to 11% [2, 3]. It can metastasize to the regional lymph nodes, central nervous system (CNS) and distant organs usually the bone and bone marrow and very rarely to the soft tissue. Here, we report a case of unilateral retinoblastoma in a 4-year-old girl accompanied by a large metastasis of the parotid and submandibular glands.

## Case presentation

**Fig. 1:**
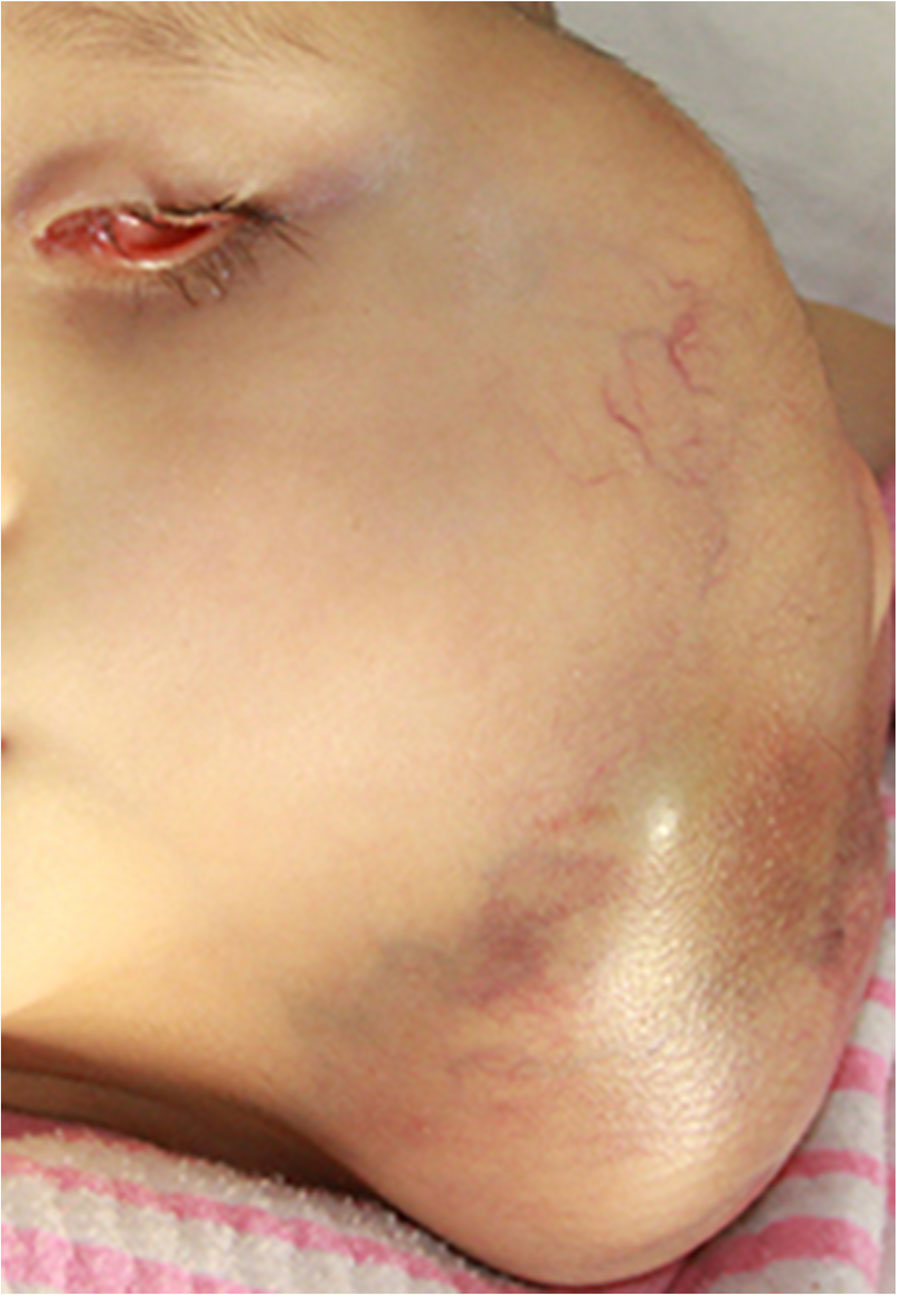
The child with left-sided facial swelling

**Fig. 2:**
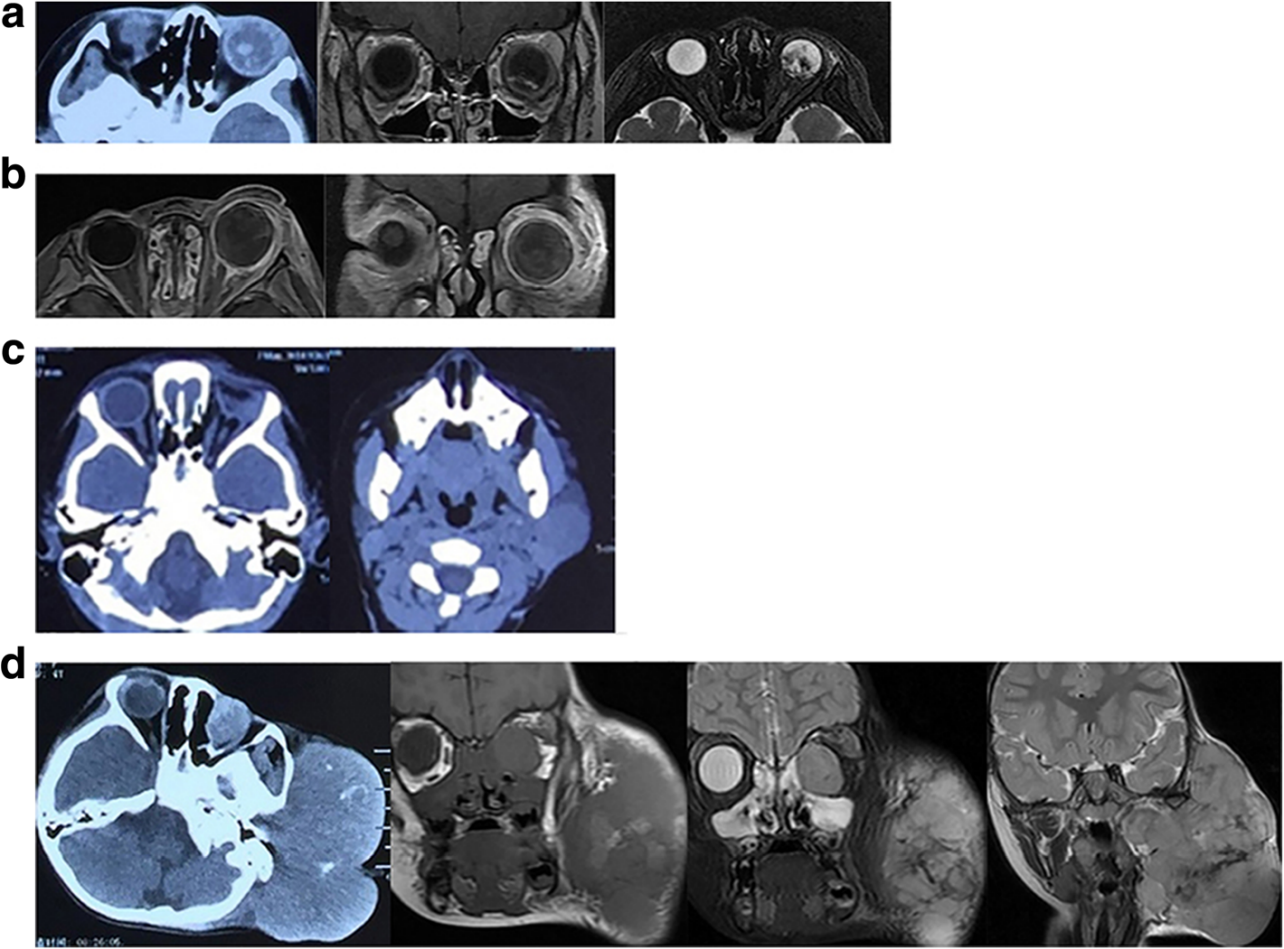
**a**: (Dec. 2012) Axial orbital CT scan shows a heterogeneous intraconal mass lesion with small foci of hyperdensity. MRI T1 scan shows a hyperintense lesion in the posterior part of the left globe that is hypointense in T2 MRI image. **b**: (Nov. 2013) MRI scan showing the larger left eye and mass outside of the left orbit. (Compare with MRI scan in Fig. 2a). **c**: (May 2014) CT scans show tumour regression in the left orbit and a small moderately intense mass on the left side of the neck with extension to the parotid and submandibular glands. **d**: (Dec. 2014) Axial CT scans show attenuation to muscle and an ill-defined solitary mass in the left orbit as well as a very large and moderately intense mass on the left side of the neck with extension to the parotid and submandibular glands. MRI scan shows iso-intensity on T1-weighted images with moderate to marked enhancement post-contrast with fat suppression, hyper-intensity on T2-weighted images in the left orbit and a large mass that was iso-intense on T1-weighted images with moderate to marked enhancement

A 4-year-old girl was admitted to our hospital in November 2014 with a large, painful and worsening mass (about 20 × 23 cm) on the left side of the neck (Fig. 1). From her past medical history, she initially presented in December 2012 (then aged 2) with a 1-month history of the parents noticing a red left eye and decreased vision. She was found to have cells in the anterior chamber, iris nodules, vitreous opacities and secondary glaucoma. The right eye was normal. Computed tomography (CT) scans showed left intraocular soft tissue density mass lesions with specks of calcifications. Magnetic resonance imaging (MRI) showed a left intraocular mass that was hyperintense relative to the vitreous on T1WI and markedly hypointense relative to the vitreous on axial T2WI (Fig. 2a). Depending on the calcification, demonstrated well with contrast administration and thin sections with fat suppression, a diagnosis of retinoblastoma stage E was made. There was no positive family history. Enucleation was proposed but was refused by the girl’s parents. After 1 year (in December 2013), the patient presented with periocular oedema and conjunctival chemosis for 3 months. She had been enucleated in another hospital. The CT and MRI before operation are shown in Fig. 2b. Histopathological examination revealed the retinoblastoma and showed that tumour cells had invaded the sclera, but tumour cells were not found at the end of the optic nerve. She did not receive any chemical or radiological treatment after the operation. In May 2014, the patient presented with a growing mass in the left orbit and small hard lump of the left side of the neck (Fig. 2c). Fine needle aspiration biopsy (FNAB) of the lump was performed, and it was found positive for tumour deposits of retinoblastoma cells in a local hospital. The parents refused chemotherapy once more due to poor financial situation. After 6 months, the patient presented again because the mass of the left side of the neck was growing too large and painful to tolerate (Fig. 2d). A multidepartment collaboration mode of treatment was applied, which involved surgery, postoperative chemotherapy and/or additional local radiotherapy.

A systemic workup including bone marrow biopsy and cerebrospinal fluid (CSF) analysis was also carried out. The bone marrow was negative for tumour deposits of malignant round cells. The CSF cytology was within normal limits. Based on this, a diagnosis of stage IVa metastatic retinoblastoma (a distant metastatic retinoblastoma without CNS involvement) was made. The systemic condition of the child was poor.

A one-stage surgery was conducted immediately. The orbital tumour was completely resected in December 2014. Since the patient had a large tumour invasion range in the left side of the neck not suitable for complete resection, the tumour was resected as much as possible while retaining organ functions. Histopathological examination revealed orbital retinoblastoma and a large intraparotid mass. The immunohistochemical staining results were as follows: Syn(++), β-micprotin(++), Ki-67(++, index 80%), CD56(++), NSE(++), Rb(++), MyoD1(++), Fli-1(+), S-100(-), CgA(-), CD99(-), CD57(-), vimentin(-), EMA(-), myogenin(-), Pax-5(-), CK18(-), LCA(-), and desmin(-). Metastasis of the parotid and submandibular glands was confirmed, and the patient underwent systemic evaluation for any other site of metastasis; however, there was no other organ involvement.

**Fig. 3:**
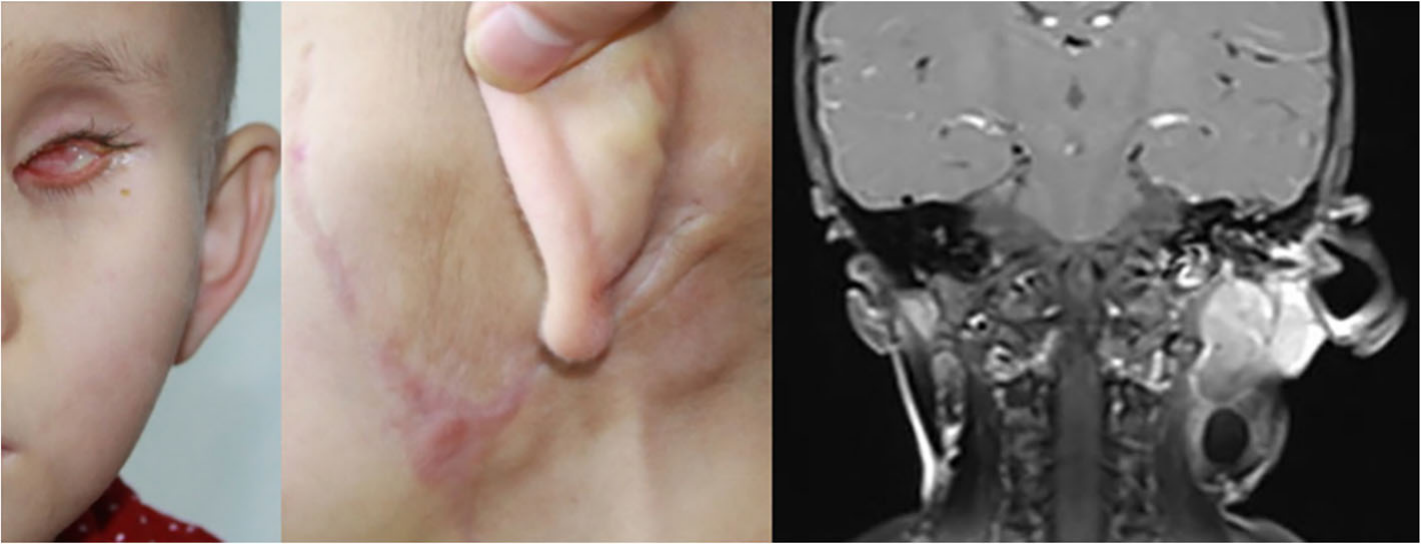
(Oct. 2016) Clinical photograph of the girl after treatment. MRI scan shows a small residual tumour in the left side of the neck. The mass seems to be hyperintense on T1 images

**Fig. 4:**
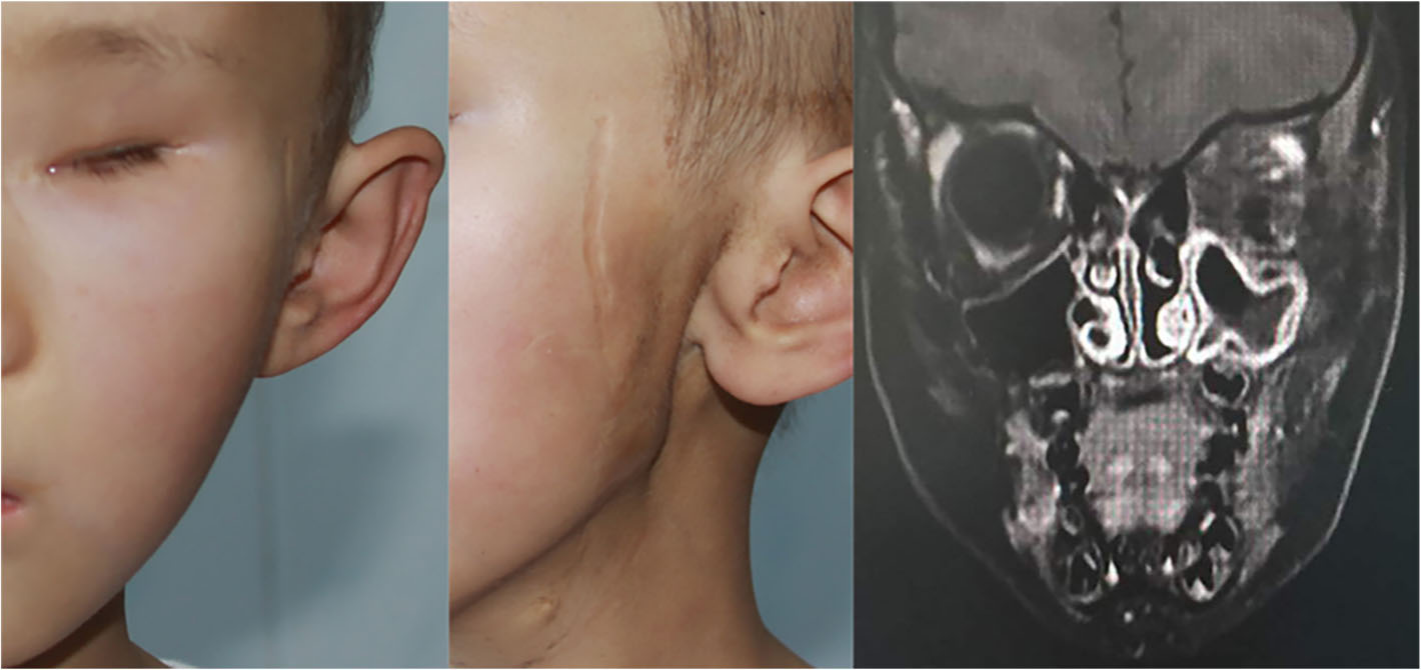
(April 2017) Clinical photograph of the girl at 28 months after operation. MRI scan shows no clear change compared with Fig. 3

The patient was started on a 3-weekly high-dose chemotherapy protocol with vincristine, etoposide and carboplatin (VEC regime). Following six cycles, the left orbital tumour had regressed completely, there was a small residual tumour in the left side of the neck, and additional local radiotherapy was performed on the parotid and submandibular glands (Fig. 3). At the most recent follow-up at 28 months, the tumour was inactive and stable as of April 2017 (Fig. 4). The girl remained on maintenance therapy and under our follow-up care.

## Discussion

Retinoblastoma is the most commonly diagnosed malignancy in children. The worldwide incidence of retinoblastoma has been reported to be 1 in 18,000 to 30,000 live births. It accounts for 3% of all childhood malignant tumours in developed countries. Metastasis of retinoblastoma is also rare in the developed world, with a reported incidence ranging from 4.8% in the United States to 5.8% in the United Kingdom, where early diagnosis achieves a cure for most children [6]. However, advanced and metastatic tumours occur frequently in developing countries, which have incidence rates of about 9 to 11%, and referral may account for delayed diagnosis [7–9]. After India, China has the most new patients with retinoblastoma [10, 11].

Retinoblastoma can metastasize to the regional lymph nodes, CNS and abdominal organs. The most common site of metastasis is the intracranial region, accounting for approximately 50% of cases [12]. Bone is a preferred secondary site. The distant bones most commonly affected by retinoblastoma metastasis are the ribs and vertebrae, which are the most active in the haematopoietic system, especially in children [13]. Retinoblastoma metastasis to the contralateral orbit, maxilla and mandible has been reported in previous articles [14, 15]. Metastasis to the soft tissue is very rare. Rare metastases to the ovaries, extremities, shoulder soft tissue, forehead and scalp have also been reported [16–18]. Retinoblastomas are known to metastasize to parotid and submandibular nodes via lymphatics. Our case shows a large intraparotid mass which has extended to the submandibular level. There have only been five previously reported cases of retinoblastoma metastasizing to parotids [19–22]. Soni et al. [19] reported the first ever case in 1978, and our case is the sixth such case reported to date.

Jubran et al. suggested four patterns of metastatic retinoblastoma, each having different clinical features and outcomes: trilateral retinoblastoma, regional metastasis, extension into CNS and distant metastasis [23]. Carbajal described five routes of metastasis: (1) cerebrospinal fluid; (2) blood stream; (3) orbit to orbit via the optic nerve; (4) contiguity directly adjacent tissue; and (5) lymph nodes [24]. In our case, metastasis to the parotid and submandibular glands and the orbit may have occurred via lymphatic and scleral invasion, respectively.

Optic nerve involvement is the most important risk factor for CNS metastasis, whereas extrascleral invasion is reported to be the most significant risk factor for distant metastasis as the tumour gains access to vascular and lymphatic channels outside the eye [3]. Other risk factors that are linked to metastasis include massive choroidal invasion, anterior segment seeding and iris and ciliary body infiltration [4, 5]. Therefore, there is a serious need for pathologic examination of the optic nerve posterior to the lamina cribrosa careful of the extirpative eyeball (particularly if there is tumour tissue at the surgical resection margin), anterior eye segment (AES) or extensive invasion of the ocular coats (massive choroidal and scleral invasion) [25–28]. The systemic workup for suspected metastatic disease includes brain and orbital MRI with and without contrast, lumbar puncture for CSF cytology, abdominal CT, bone scan and bone marrow biopsy. The laboratory workup includes complete blood count with differential count, liver function test, renal function test and serum lactate dehydrogenase measurement. In our case, the results of the systemic workup were within normal limits, except in the orbit and the left side of the neck.

A definitive cure for intraocular retinoblastoma with no possibility of useful vision is achieved by removal of the eye before the tumour spreads [11, 29]. However, the management of extraocular disease is complex. Chemotherapy was advised after operation to reduce the metastasis. When the tumour extends beyond the globe into the orbit or distant metastasis, a combination of radiation therapy and high-dose chemotherapy ought to be used. High-dose (3-weekly, 6-12 cycles): Vincristine 0.025 mg/kg, Etoposide 12 mg/kg, Carboplatin 28 mg/kg [30]. In our case, the left orbital tumour regressed completely after six cycles, and only a small residual tumour remained on the left side of the neck. Additional local radiotherapy was then performed on the small residual tumour. At the most recent follow-up at 28 months, the patient was stable.

The prognosis remains relatively poor for patients whose disease disseminates into the CNS and those with distant metastatic disease [31]. There is a lot of new, encouraging literature suggesting that high-dose chemotherapy with autologous stem cell rescue is associated with improved survival for patients with distant metastatic retinoblastoma without CNS involvement, as in our case [32]. Antoneli CB, M.D., Ph.D., showed that bone marrow transplantation is used in patients with metastatic retinoblastoma and appears to be effective and that ophthalmic artery chemosurgery (OAC) is also used in retinoblastoma patients with second primary malignancies, although to our knowledge, the number of reported successful cases is still small enough to recommend this procedure as the gold standard of treatment in these patients [9, 33]. In many cases, various combinations of treatment may be needed to achieve a satisfactory result. Children’s oncology group trials may help formulate a relatively effective strategy for the management of metastatic retinoblastoma, which remains a challenge in paediatric oncology.

## Conclusions

A definitive cure for intraocular retinoblastoma with no possibility of useful vision is achieved by removal of the eye before the tumour spreads. Additional adjuvant chemotherapy and radiotherapy followed by appropriate surgery are advised after operation to reduce metastasis. When the tumour extends beyond the globe into the orbit or metastasizes to a distant site, a combination of radiation therapy and high-dose chemotherapy should be used. Delayed detection and inappropriate management contribute to poor outcomes. Fundus examinations, education regarding the early signs of RB, and optimization of the therapeutic strategy for RB may play important roles in ocular health. Early detection, accurate diagnosis, and active intervention are conducive to control of retention of patients’ vision. A better referral system and/or an education programme for referring physicians are needed to prevent such cases in the future.

## Abbreviations

CNS: Central nervous system
CSF: Cerebrospinal fluid
CT: Computed tomography
MRI: Magnetic resonance imaging
